# Predictive value of 1H MR spectroscopy and 18F-FDG PET/CT for local control of advanced oropharyngeal and hypopharyngeal squamous cell carcinoma receiving chemoradiotherapy: a prospective study

**DOI:** 10.18632/oncotarget.23306

**Published:** 2017-12-14

**Authors:** Chih-Hua Yeh, Gigin Lin, Jiun-Jie Wang, Chien-Yu Lin, Shang-Yueh Tsai, Yu-Chun Lin, Yi-Ming Wu, Sheung-Fat Ko, Hung-Ming Wang, Sheng-Chieh Chan, Tzu-Chen Yen, Chun-Ta Liao, Shu-Hang Ng

**Affiliations:** ^1^ Department of Medical Imaging and Intervention, Chang Gung Memorial Hospital, Linkou, Chang Gung University, Taoyuan, Taiwan; ^2^ Molecular Imaging Center, Chang Gung Memorial Hospital, Linkou, Taoyuan, Taiwan; ^3^ Department of Medical Imaging and Radiological Sciences, Chang Gung University, Taoyuan, Taiwan; ^4^ Clinical Phenome Center and Imaging Core Lab, Institute for Radiological Research, Chang Gung Memorial Hospital, Linkou, Taoyuan, Taiwan; ^5^ Department of Diagnostic Radiology, Chang Gung Memorial Hospital, Keelung, Taiwan; ^6^ Department of Radiation Oncology, Chang Gung Memorial Hospital, Linkou, Chang Gung University, Taoyuan, Taiwan; ^7^ Graduate Institute of Applied Physics, National Chengchi University, Taipei, Taiwan; ^8^ Department of Diagnostic Radiology, Chang Gung Memorial Hospital, Kaohsiung, Taiwan; ^9^ Department of Medical Oncology, Chang Gung Memorial Hospital, Linkou, Chang Gung University, Taoyuan, Taiwan; ^10^ Department of Nuclear Medicine, Chang Gung Memorial Hospital, Linkou, Chang Gung University, Taoyuan, Taiwan; ^11^ Department of Otorhinolaryngology, Head and Neck Surgery, Chang Gung Memorial Hospital, Linkou, Chang Gung University, Taoyuan, Taiwan

**Keywords:** oropharyngeal cancer, hypopharyngeal cancer, MR spectroscopy, positron emission tomography, chemoradiotherapy

## Abstract

**Purpose:**

To determine whether pretreatment *in vivo* 1H magnetic resonance (MR) spectroscopy at 3 Tesla (T) and 18F-FDG PET/CT can offer predictive power regarding the local control of oropharyngeal or hypopharyngeal squamous cell carcinoma (OHSCC) patients.

**Materials and Methods:**

^1^H MR spectroscopy was performed in addition to conventional MR imaging before definitive chemoradiotherapy in 58 patients with advanced OHSCC. The relationship of local control with the ^1^H MR spectroscopy and ^18^F-FDG PET/CT parameters was analyzed.

**Results:**

With a median follow-up of 17.6 months, 13 (22.4%) patients exhibited local failure; whereas the remaining 45 achieved local control. Kaplan-Meier analysis identified age > 60 years, creatine level on MRS ≦ 6.02 *mM*, glutamine and glutamate (Glx) level on MRS > 3.31 *mM*, and total lesion glycolysis (TLG) on ^18^F-FDG PET/CT > 217.18 *g/mL × mL* as significant adverse predictors for 2-year local control. Multivariate Cox regression analysis showed that age (p=0.017), Glx level on MRS (p=0.021), and TLG on ^18^F-FDG PET/CT (p=0.028) retained their independent prognostic significance. A scoring system was constructed based on the sum of these three factors. We found that patients with scores of 2–3 had significantly poorer local control rates than patients with scores of 0–1 (33.3% versus 86.8%, p=0.003).

**Conclusion:**

We conclude that Glx on ^1^H MR spectroscopy at 3 T was the independent prognostic factor for local control of OHSCC patients treated with chemoradiotherapy, and its combination with age and TLG may help identify a subgroup of patients at high risk for developing local failure.

## INTRODUCTION

Oropharyngeal and hypopharyngeal squamous cell carcinoma (OHSCC) are common cancers of the head and neck region. These neoplasms have contiguous anatomical origins and share similar lymphatic drainage and treatment regimens. OHSCC usually manifests as the advanced disease (stage III-IV) at presentation and is primarily treated with an organ-preservation approach based on definitive chemoradiotherapy [1, 2]. However, local failure remains a significant problem, with the reported local failure rates ranging from 20% to 30% [2–5]. Early prediction of treatment failure may enable therapeutic modification, including earlier salvage surgery for suitable cases and upfront intensification of chemotherapy or radiotherapy [6]. Therefore, the identification of reliable imaging biomarkers predicting treatment effects will enable improved prognosis stratification of patients undergoing chemoradiotherapy.

While computed tomography (CT) and magnetic resonance imaging (MRI) are commonly used to evaluate tumor extent of head and neck squamous cell carcinoma (HNSCC), MRI is more preferred in the delineation of anatomical tumor extent in the OHSCC by its high soft tissue contrast resolution. Proton magnetic resonance (^1^H MR) spectroscopy is an advanced MRI technique with a potential to relate molecular biochemistry to the biological behavior of normal and malignant tissues. ^1^H MR spectroscopy has been introduced clinically in patients with HNSCC, with choline being one of the most important metabolites [7–11]. Mukherji et al. reported that the choline/creatine ratios in primary tumors and their metastatic nodes were significantly elevated above those in normal muscle structures [7]. Bisdas et al. [9] have found that percentage changes in choline levels following chemoradiotherapy helped differentiate recurrent disease from post-therapeutic inflammatory tissues. However, King et al. [11] revealed that the presence of choline in a post-treatment mass, instead of its percentage changes, may serve as a marker of residual cancer. With the *development* in ^1^H MR spectroscopy technology, magnetic field strength and quantification modeling, some other metabolites including lipids, glutamine and glutamate (Glx), and myo-inositol have been found to elevate in tissue specimens of HNSCC [7, 12–15]. However, whether these metabolites can be identified using *in vivo* high magnetic field MR spectroscopy, and to what extent they help prediction of tumor control of OHSCC patients undergoing chemoradiotherapy have not been investigated.

Nowadays, [^18^F]-fluorodeoxyglucose positron emission tomography/CT (^18^F-FDG PET/CT) has become increasingly popular in the evaluation OHSCC because it provides anatomical and functional information for the entire body. Its quantitative parameters, including maximum standardized uptake value (SUV_max_), metabolic tumor volume (MTV), and total lesion glycolysis (TLG) have been used to correlate with tumor control of HNSCC after chemoradiotherapy; however, variable results have been reported [5, 16–22]. SUV_max_ has been identified to be the independent predictor [5, 16–18]. However, some other studies observed that SUV_max_ was not related to treatment response, whereas MTV [19, 21], TLG [22], or both [20] were found to be adverse prognostic factors for local failure. More recently, the measurement of texture indices from tumor PET images has also been proposed to show the FDG uptake distribution within the tumor. In the post-hoc analysis of 124 cases collected from previous research projects [23], we demonstrated that FDG uptake heterogeneity was superior to traditional PET parameters in prognostic stratification of advanced OHSCC patients treated with chemoradiotherapy. Accordingly, we conducted a prospective study to determine whether pretreatment *in vivo* ^1^H MR spectroscopy at 3 Tesla (T) and ^18^F-FDG PET/CT can offer predictive power regarding the local control of advanced OHSCC patients treated with definitive chemoradiotherapy.

## RESULTS

Between August 2013 and September 2015, a total of 77 OHSCC patients underwent MR spectroscopy and ^18^F-FDG PET/CT. Nineteen patients were excluded from the analysis, 16 of whom CRLB values of methyl resonance at 1.3 parts per million (ppm) exceeding the 30% range, and 3 died before the definitive diagnosis of recurrence could be made. Therefore, a total of 58 patients were eligible for the analysis. Male were predominant (N=56, 96.5%) with the median age 53.8 years. The primary tumor sites consisted of 31 (53.5%) oropharynx and 27 (46.5%) hypopharynx, and all were in advanced T categories with 14 (24.1%) T3, 38 (65.6%) T4a and 6 (10.3%) T4b. Patient characteristics are summarized in Table 1. All patients underwent definitive chemoradiotherapy and were closely monitored. The median follow-up time for the entire cohort was 17.4 months and 20.1 months for the survivors. In posttreatment 3-month MRI follow-up, complete response of the primary tumor was noted in 47 of our 58 (81%) patients, while 7 (12.1%), 2 (3.5%), and 2 (3.5%) patients had partial response, stable disease, and progressive disease, respectively, based on the RECIST criteria. The volume changes of our seven patients of partial response ranged from −72.7% to −87.3% (median: −82.9%). The volume changes of our two patients with progressive disease were 187.2% and 193.6%. respectively. At the end of observation, 36 (62.1%) patients were alive, and 22 (37.9%) were dead. Forty-five (77.6%) patients achieved durable local control, whereas 13 (22.4%) patients had local failures (pathological confirmation, N=7; disease progression in serial imaging studies, N=6). The 2-year local control rate and overall survival rate were 54.9% and 72.1%, respectively.

**Table 1:** Demographic information of enrolled patents (n = 58)

Characteristic	n (%)
Sex	
Male	56 (96.6)
Female	2 (3.4)
Age, years, median (range)	53.8 (30–79)
Tumor site	
Oropharynx	31 (53.4)
Hypopharynx	27 (46.6)
Pathologic differentiation	
Well	2 (3.4)
Moderate	30 (51.7)
Poor	16 (27.6)
N/A	10 (17.2)
Tumor status	
T3	14 (24.1)
T4a	38 (65.6)
T4b	6 (10.3)
Nodal status	
N0	5 (8.6)
N1	7 (12.1)
N2a	0 (0.0)
N2b	26 (44.8)
N2c	12 (20.7)
N3	8 (13.8)
Stage	
III	5 (8.6)
IVA	41 (70.7)
IVB	12 (20.7)

N/A: not available.

Pearson’s correlation analysis revealed that tumor size was *modestly* correlated with levels of lipid methylene (1.3 ppm) and levels of lipid unsaturated fatty acyls (2.0 ppm) on MR spectroscopy (correlation coefficients = −0.272 and −0.260, and p = 0.039 and 0.049 respectively), as well as with TLG and MTV (correlation coefficients = 0.706 and 0.690 respectively, and p both < 0.001) on ^18^F-FDG PET/CT. Among the metabolites detected on MR spectroscopy, choline level was significant correlated with creatine level *(p < 0.001)* and myo-inositol level *(p = 0.003)*. The resonance level of Glx was found to have significant correlation with lipids levels at 1.3 and 2.0 ppm *(p both <0.001)*. Lipid methylene (1.3 ppm) level was *modestly* correlated with TLG *(p = 0.05)* and MTV *(p = 0.048)*, while Glx level did not correlate with any ^18^F-FDG PET/CT parameters.

**Figure 1:**
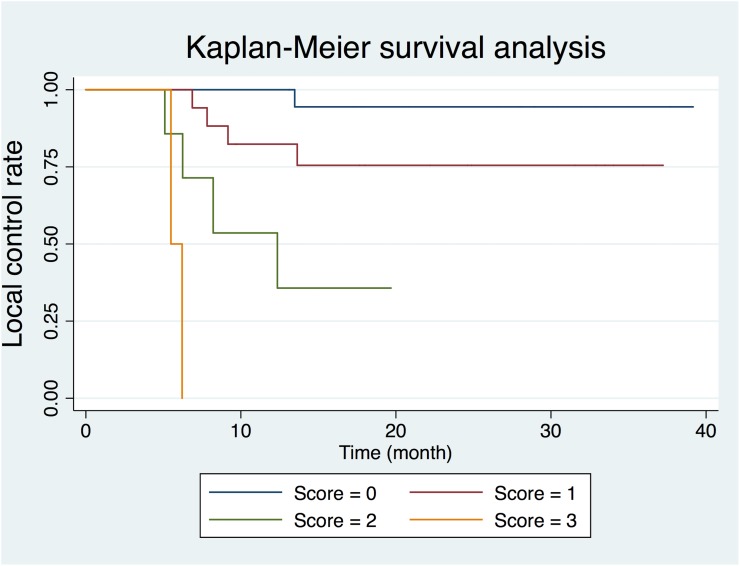
Kaplan-*Meier* plot based on the risk score generated by age, glutamine and glutamate (Glx) on MR spectroscopy, and total lesion glycolysis on ^18^F-FDG PET/CT

Log-rank test of Kaplan-Meier survival analysis for 2-year local tumor recurrence revealed that age > 60 years (p=0.041), creatine level ≦ 6.02 *mM* on MR spectroscopy (p = 0.041), Glx level > 3.31 *mM* on MR spectroscopy (p = 0.001) and TLG > 402.24 *g/mL × mL* on ^18^F-FDG PET/CT (p = 0.002) were significantly correlated with poor 2-year local control rate (Table 2). The remaining variables, including age, tumor site, T status, N status, tumor size, creatine, choline, myo-inositol, lipid methyl (0.9 ppm), lipid methylene (1.3 ppm), and lipid unsaturated fatty acyls (2.0 ppm), SUV_max_, and MTV, showed no significant impacts on local control. Univariate Cox regression analysis also identified Glx (p = 0.005) and TLG (p = 0.005) as significant predictors of 2-year local control rate, and age (p = 0.053) as borderline significant predictor of 2-year local control. In multivariate Cox regression, age (p = 0.017), Glx (p = 0.021), and TLG (p = 0.028) remained as significant independent predictors of 2-year local control (Table 3). We further assessed whether combination of these three independent predictors could help predict local control of OHSCC after chemoradiotherapy. A prognostic scoring system was constructed based on the sum of the three predictors, including age > 60 years, Glx level on MR spectroscopy > 3.31 *mM*, and TLG level on ^18^F-FDG PET/CT > 402.24 *g/mL × mL*. The presence of each risk factor assigned a score of 1 while an absence of a risk factor scored 0, resulting in a total score of 0, 1, 2 or 3. It was found that patients with cumulated scores of 0, 1, 2 and 3 differed significantly regarding 2-year local control rates (95.0%, 77.8%, 42.9% and 0%, respectively, Figure 1). Patients with scores of 0–1 had significantly better 2-year local control rates than patients with scores of 2–3 (86.8% versus 33.3%, p=0.003). Multivariate Cox proportional hazard analysis demonstrated a poorer local control rate in patients with a score of 1–2 than those with a score of 0 (hazard ratio [HR] = 5.220 and 21.494; p = 0.140 and 0.006, respectively), while patients with a score of 3 showed the poorest local control rate (HR = 314.834; p < 0.001) (Table 4).

**Table 2:** Kaplan-Meier analysis of parameters and two-year local control rates

Parameters	n	2-year localcontrol rate (%)	p value
Age (years)			0.041
≤ 60	45	82.2	
> 60	13	17.8	
Primary tumor site			0.651
Oropharynx	31	80.6	
Hypopharynx	27	74.1	
T status			0.323
T_3_	14	85.7	
T_4_	44	75.0	
N status			0.855
N_0-1_	12	75.0	
N_2-3_	46	78.3	
Tumor size (cm)^#^			0.095
≤ 4.71	25	88.0	
> 4.71	33	69.7	
Creatine (mM)^*^			0.041
≤ 6.02	36	69.4	
> 6.02	22	90.9	
Choline (mM)^*^			0.111
≤ 3.33	38	73.7	
> 3.33	20	85.0	
Myo-Inositol (mM)^*^			0.219
≤ 6.05	34	82.4	
> 6.05	24	70.8	
Glx (mM)^*^			0.001
≤ 3.31	34	88.2	
> 3.31	24	62.5	
Lipid methyl (δ 0.9 ppm, mM)^*^			0.772
≤ 0.093	27	81.5	
> 0.093	31	74.2	
Lipid methylene (δ 1.3 ppm, mM)^*^			0.205
≤ 734.31	47	74.5	
> 734.31	11	90.9	
Lipid unsaturated (δ 2.0 ppm, mM)^*^			0.342
≤ 0.016	23	73.9	
> 0.016	35	80.0	
Standardized uptake value (g/mL)^+^			0.154
≤17.28	25	72.0	
>17.28	31	80.6	
Metabolic tumor volume (mL)^+^			0.092
≤ 18.07	15	93.3	
> 18.07	41	70.7	
Total lesion glycolysis (g/mL × mL)^+^			0.002
≤ 402.24	42	83.3	
> 402.24	14	57.1	

^#^ Measurement based on gadolinium-enhanced fat-saturated T1-weighted MR images.

^*^ Measurement based on MR spectroscopy.

^+^ Measurement based on 18F-Fluorodeoxyglucose-positron emission tomography.

Glx: glutamine and glutamate; ppm: parts per million; mM: millimole.

**Table 3:** Cox regression analysis of parameters associated with 2-year local control rate

Local failure (n=13)	Univariate analysis			Multivariate stepwise		
Parameter	HR	95% CI	p value	HR	95% CI	p value
Age (> 60 vs ≤ 60)	3.246	0.986 - 10.692	0.053	4.823	1.327 - 17.536	0.017
Primary tumor site(hypopharynx vs oropharynx)	1.314	0.400 - 4.310	0.652			
T status (T_4_ vs T_3_)	2.131	0.459 - 9.890	0.334			
N status (N_2-3_ vs N_0-1_)	0.884	0.234 - 3.337	0.855			
Tumor size^#^	2.950	0.779 - 11.165	0.111			
Creatine^*^	0.155	0.020 - 1.213	0.076			
Choline^*^	0.219	0.028 - 1.710	0.148			
Myo-Inositol^*^	2.075	0.632 - 6.808	0.229			
Glx^*^	8.954	1.926 - 41.619	0.005	6.449	1.319 - 31.519	0.021
Lipid methyl (δ 0.9 ppm)^*^	1.191	0.363 - 3.907	0.773			
Lipid methylene (δ 1.3 ppm)^*^	0.287	0.037 - 2.249	0.235			
Lipid unsaturated (δ 2.0 ppm)^*^	0.567	0.173 - 1.857	0.348			
Standardized uptake value+	0.420	0.123 - 1.436	0.167			
Metabolic tumor volume+	4.922	0.629 - 38.492	0.129			
Total lesion glycolysis+	5.861	1.685 - 20.387	0.005	4.840	1.183 - 19.811	0.028

^#^ Measurement based on gadolinium-enhanced fat-saturated T1-weighted MR images.

^*^ Measurement based on MR spectroscopy.

^+^ Measurement based on 18F-Fluorodeoxyglucose-positron emission tomography.

Glx: glutamine and glutamate; HR: hazard ratio; CI: confidence interval; ppm, parts per million.

**Table 4:** Risk score based on Glx on MR spectroscopy and total lesion glycolysis on 18F-FDG PET/CT

Score	No. of patients	HR	95% CI	p value
0	23	1.000	Reference	
1	22	5.220	0.583 - 46.770	0.140
2	10	21.494	2.375 - 194.554	0.006
3	3	314.834	14.268 - 6947.230	< 0.001

Glx: glutamine and glutamate; HR: hazard ratio; CI: confidence interval.

## DISCUSSION

Our results demonstrated the value of pretreatment ^1^H MR spectroscopy at 3 T and ^18^F-FDG PET/CT for patients with advanced OHSCC for predicting local control following chemoradiotherapy. Elevated Glx levels on ^1^H MR spectroscopy, old age and high TLG on ^18^F-FDG PET/CT were adversely correlated with the 2-year local control rate. Glutamine is the most abundant amino acid in the body and can be an essential nutrient for cell growth and viability. It is a major anaplerotic substrate in proliferating cells by conversion into α-ketoglutarate for biosynthesis. Independent lines of investigation have demonstrated that cancer cells may be addicted to glutamine [24–26]. Elevation of Glx levels due to glutamine scavenge of tumors is supported by several *ex vivo* ^1^H MR spectroscopy studies from investigating tissue samples [12–14, 24–26], primarily for differentiating cancer from normal tissues. Yuneva et al. [26] reported that some proto-oncogene MYC transformation were dependent on glutamine, and understanding the linkage of glutamine metabolism to cell viability may provide new insights for cancer treatment. In a study exploring the metabolic signatures of HNSCC, Somashekar et al. [13] reported that elevated levels of Glx and choline-containing compounds were found in HNSCC tissues, suggesting their association with active glycolysis, increased amino acids influx into the tricarboxylic acid cycle, altered energy metabolism, and membrane choline phospholipid metabolism. To our knowledge, our study is the first *in vivo* ^1^H MR spectroscopy investigation to demonstrate elevated Glx resonance levels in OHSCC patients as a possible adverse prognostic factor.

Choline and its derivatives are important constituents in phospholipid metabolism of cell membranes and identified as markers of cell proliferation. Previous *in vitro* and *in vivo* ^1^H MR spectroscopy studies showed that the choline-to-creatine ratio was significantly higher in HNSCC than in normal tissue or post-therapeutic tissue [7, 9]. In a study by Bezabeh et al. [8], significantly elevated choline-to-creatine ratio was observed in tumor tissue specimens of HNSCC patients with treatment failure, suggesting that *ex vivo* ^1^H MR spectroscopy of tissue samples has the potential to predict response to the treatment in HNSCC. *in vivo* ^1^H MR spectroscopy studies of HNSCC with 1.5T MR scanner, King et al reported that both the pretreatment choline and change in choline early during a course of treatment did not predict clinical outcome [10], while the presence of choline in a post-treatment mass, instead of a percentage change in choline ratios, may serve as a marker of residual cancer [11]. In the present prospective *in vivo* ^1^H MR spectroscopy study of OHSCC using a 3 T MR scanner, the pretreatment tumor not choline level did not demonstrate prognostic value for tumor control after chemoradiotherapy.

Myo-inositol is a precursor in the phosphatidylinositol cycle and a source of several second messengers. Myo-inositol is involved in the activation of protein C kinase. Protein C kinase leads to the production of protylotic enzymes, which are found more often in malignant and aggressive primary tumors. Elevation of myo-inositol may occur either by rapid cellular proliferation or cellular distraction [27]. Previous studies have showed that myo-inositol acts as a cancer chemoprevention agent [28, 29]. On the other hand, lipids function as energy storage molecules, structural components of cell membranes, and signaling molecules involved in cell growth. Increased lipid biosynthesis is a characteristic feature of cancer [30]. In an *in vivo* ^1^H MR spectroscopy study at 3 T, Lin et al. [15] identified elevated lipid resonance levels in uterine cervical carcinomas, which help to predict persistent disease following chemoradiotherapy. However, in the present study, neither myo-inositol nor lipid resonance levels had prognostic value in local tumor control of OHSCC after chemoradiotherapy.

It is difficult to explain why Cho, myo-inositol and lipid resonance levels did not demonstrate significant predictive values in our study. We thought that some of our OHSCC cases presented with intratumoral heterogeneity, in terms of cellular proliferation, vascularity or necrosis, contributing to variable results about Cho, myo-inositol and lipid metabolites on MR spectroscopy. Therefore, it should be cautious to use MR spectroscopy alone to predict prognosis of OHSCC. MR spectroscopy should be viewed together with other parameters, being age and TLG in our cases.

^18^F-FDG PET/CT, an imaging modality that incorporates both anatomical localization and functional information, and it is now increasingly used for pretreatment evaluation of OHSCC patients. It can provide three quantitative parameters, including SUV which reflects glucose utilization, MTV which reflects tumor burden, and TLG which integrates both glucose utilization and tumor burden. However, the prognostic significance of these parameters in HNSCC patients still remains controversial. Pretreatment SUV_max_ of ^18^F-FDG PET has been reported to be an independent prognostic factor of local control in HNSCC treated by radiotherapy or chemotherapy [5, 16–18]. However, some other studies showed that MTV and TLG, instead of SUV_max_, were adverse prognostic factors for local failure [19–22]. In the present study, we found that tumor TLG had a better prognostic value than SUV_max_ or MTV, consistent with those studies showing the superior usefulness of tumor TLG in predicting treatment outcome in oropharyngeal SCC [20, 22]. This can be plausibly explained by tumor TLG incorporating both metabolic activity and active tumor volume and, hence, better reflection of tumor characteristics. Indeed, our data also revealed that T stage and tumor size were not significant prognostic parameters. T stage for OHSCC takes into account only the greatest single dimension of the primary tumor (T1–T3) or infiltration of surrounding tissue (T4) based on clinical examination and morphological imaging. However, neither clinical examination nor morphological imaging can differentiate active tumor reliably from contiguous inflammatory tissue or tumor-related edema. Therefore, their size measurements might not always indicative the actual active tumor burden, and, hence, would not significantly imply the probability of local control.

The use of chemoradiotherapy has increased substantially in the elderly patients of head and neck cancer [31]. Although the addition of chemotherapy to radiation has proven efficacious in many randomized controlled trials, it may be less effective for head and neck cancer in an older patient population [32, 33]. Our patients in this prospective cohort study were uniformly treated with cisplatin-based concurrent chemoradiation therapy, and our results showed that advanced age was associated with local failure within 2 years. Furthermore, our study showed that the combination of old age with high TLG and elevated Glx levels in the primary tumor signified a subgroup of OHSCC patients at high risk of local failure after chemoradiation. An early prediction of failure in such patients may urge close surveillance of posttreated primary sites to ensure timely detection of potentially salvageable lesions, or, alternatively, may allow for therapeutic modification, including the selection of suitable candidates for surgery or trials of novel treatment approaches.

The primary limitation of the present study was that the head and neck is a technically difficult anatomical region in which to obtain high-quality MR spectra consistently because of motion and susceptibility artifacts. Sixteen of our 77 (20.7%) patients were excluded from the analysis due to suboptimal spectra. Further refinement of respiratory gating and anti-susceptibility technology is warranted. Second, the sample size was limited in this prospective study. Further study with more subjects is suggested to draw more persuasive arguments. In addition, only primary tumors with shortest axial diameters >15 mm were investigated. Moreover, we did not have smaller tumors or normal controls for comparison because interpretable MR spectra are exceedingly difficult to obtain in such thin pharyngeal walls. Finally, the sample size in this study might not large enough to draw a definite conclusion. Further large-scale studies are needed to establish the predictive role of 3T MR spectroscopy in OHSCC.

In conclusion, Glx on ^1^H MR spectroscopy at 3 T was the independent prognostic factor for local control of OHSCC patients treated with chemoradiotherapy, and its combination with age and TLG may help identify a subgroup of patients at high risk of developing local failure.

## MATERIALS AND METHODS

### Patients

The study protocol was approved by the Institutional Review Board of our Hospital. All participants provided informed written consent, and the study adhered to the tenets of Declaration of Helsinki. From August 2013 to September 2015, patients with histologically proven OHSCC, who were scheduled to receive definitive chemoradiotherapy with curative intent, were eligible for this prospective study. The study subjects underwent a thorough pretreatment evaluation, including MRI and ^18^F-FDG PET/CT. The inclusion criteria included the following: the presence of primary tumors with shortest axial diameters > 15 mm on MR images; adults aged; ability to provide informed consent; and no contraindications to MR scanning, such as claustrophobia or cardiac pacemaker. Patients with previous cancers, a synchronous malignant tumor, or distant metastasis at presentation were excluded.

All participants underwent intensity-modulated radiotherapy or volumetric modulated arc therapy using 6-MV photon beams at a daily fraction of 2 Grays (Gy), with five fractions per week. The radiotherapy dose was 46-50 Gy for all subclinical risk areas and 72 Gy for gross tumor areas. Concurrent chemotherapy consisted of intravenous cisplatin (50 mg/m^2^) on day 1, oral tegafur (800 mg/day) plus oral leucovorin (60 mg/day) from day 1 to day 14. This regimen was delivered every 14 days through the course of radiotherapy [34]. After treatment, patients underwent a routine clinical follow-up every 1 to 3 months. Posttreatment MRI was performed three months after completion of chemoradiotherapy, and follow-up MRI or CT has performed alternatively every six months after that or in response to clinical deterioration. The endoscopic biopsy was performed, if possible, for any suspicious residual/recurrent tumors. Patients without pathological-proven local failure were monitored over a minimum follow-up of 12 months after initial treatment or until death.

### MRI and MR spectroscopy

MRI was performed using a 3-T scanner (Magnetom Trio with TIM, Siemens, Erlangen, Germany) with head and neck matrix coil. All patients underwent conventional head and neck MRI before and after gadolinium-diethylenetriaminepenta-acetic acid (Gd-DTPA) injection. Unenhanced T1-weighted turbo spin echo (TSE) images (*TE ms/TR ms: 11/400 ; flip angle: 150 degree*) and T2-weighted fat-suppressed TSE images *(TE ms/TR ms: 88/5380, flip angle: 120 degree)* were acquired in the axial and coronal planes. Field of view (FOV) was 220 mm in axial projection and was 300 mm in coronal projection. After Gd-DTPA intravenous injection, T1-weighted fat-suppressed axial, sagittal, and coronal TSE sequences *(TE ms/TR ms: 11/415 ; flip angle: 130 degree)* were obtained sequentially.

After that, triplane localizer MR spectroscopy with point-resolved spectroscopy (PRESS) was performed by selecting a 12 mm × 12 mm × 12 mm spectroscopic volume prescribed by a neuroradiologist with more than 20 years’ experience in head and neck radiology. With MRI guidance, the volume of interest was meticulously placed within the primary tumor, while visually cystic or necrotic areas were avoided. The B0 shimming parameters were optimized using a rapid B0 mapping method which takes advantage of the combination of dynamic shimming and continuous frequency updates [35]. For each voxel placement, automated optimization of gradient shimming was performed. The water line width within the PRESS box was measured after shimming. If the value was > 15 Hz, the PRESS box was repositioned and re-shimmed. B0 maps (dual gradient-echo; TR 50 ms, TEs 7.6 ms, and 17.6 ms) were then obtained to ensure there was no significant B0 inhomogeneity or susceptibility artifacts. MR spectroscopy was acquired using the following parameters: TR/TE, 2000 ms/35 ms; 128 averages; vector size, 1024 points; and bandwidth, 1200 Hz. Water suppression was achieved using band selective inversion with gradient dephasing. Non-water suppressed spectra as concentration references were also measured with the same scan parameters, except with the acquisition of only four signal averages to reduce scan time to 16 second. The flip angle was 90 degree and TR was 2000 ms for both non-water suppressed and water suppressed scans. The total scan time for MR spectroscopy was approximately 7 minutes.

### ^18^F-FDG PET/CT

^18^F-FDG PET/CT scans were performed using an integrated PET/CT system (Discovery ST 16, GE Healthcare, Milwaukee, WI). The axial and transaxial fields of view (FOVs) of the PET component are 15.7 and 70 cm, respectively. The transaxial resolution is 6.1-mm full width at half maximum (FWHM) 1 cm off center. The length of the PET table is 275 cm. Patients were instructed to fast for at least 6 hours before the examination. Before PET acquisition, helical CT from the head to the proximal thigh was performed using the following parameters: 100 mA, 100 kVp, collimation 16 mm x 3 mm, tube rotation time 0.5 s, pitch 1.5, and table speed 35 mm/s. Subsequently, PET emission scans were performed between 50 min and 70 min after injection of ^18^F-FDG (370 MBq) with the coverage from the head to the proximal thigh. Images were acquired in the two-dimensional mode, with 3-min per table position. The PET emission data were reconstructed using CT data for attenuation correction using the ordered subsets expectation maximization method with 10 subsets and 4 iterations.

### Data analysis

**Figure 2:**
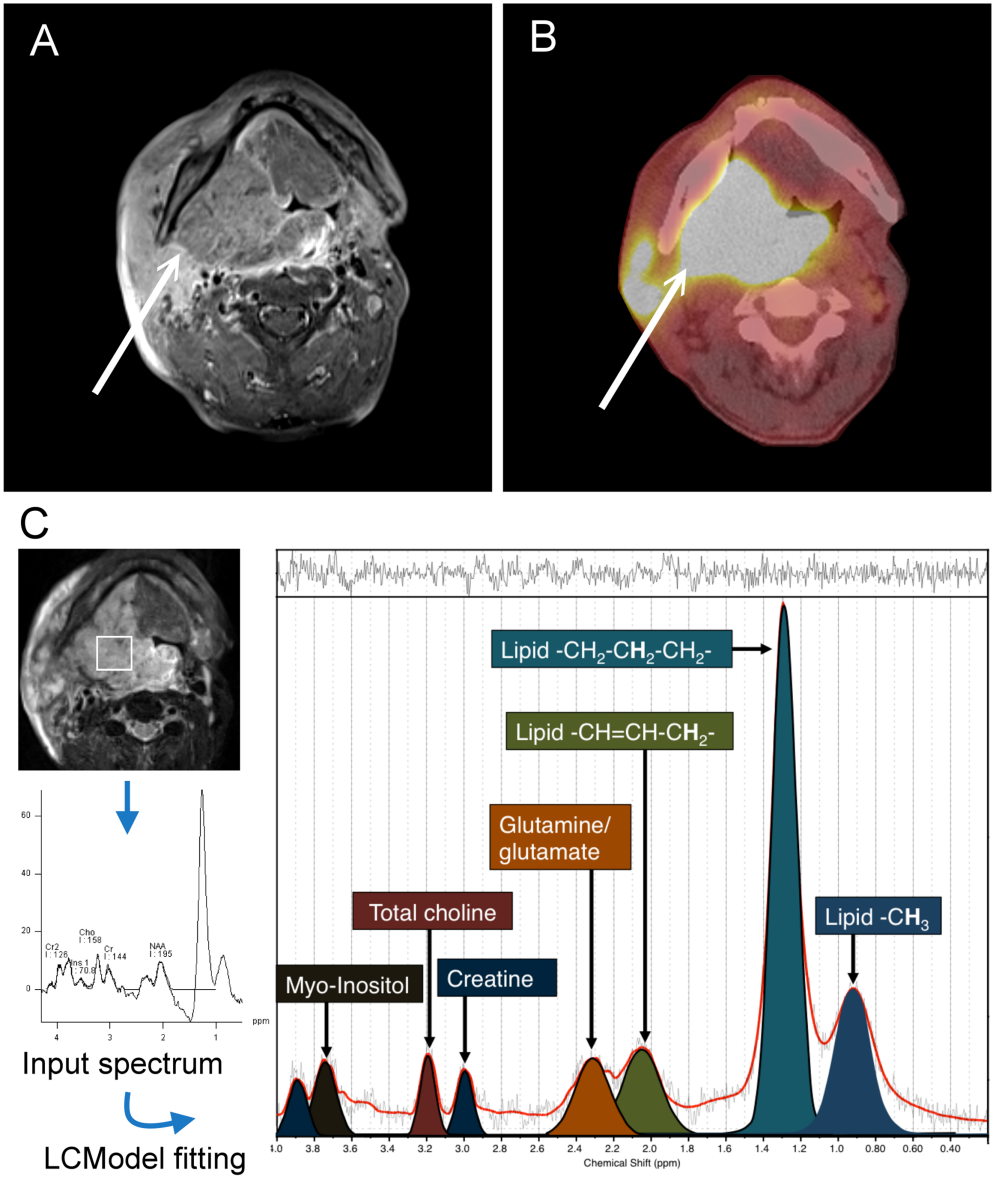
A 54-year-old man with right tonsillar squamous cell carcinoma **(A)** Axial contrast-enhanced fat-saturated T1-weighted MR image. **(B)** The corresponding ^18^F-FDG PET/CT image. **(C)** The corresponding ^1^H-MR spectrum of this subject. Resonances of lipid δ 0.9 ppm (methyl, -CH_3_), δ 1.3 ppm (-CH_2_-CH_2_-CH_2_-), δ 2.0 ppm (-CH=CH-CH_2_-), glutamate and glutamine (Glx) δ 2.2-2.4 ppm, creatine δ 3.0 and 3.9 ppm, total choline resonance δ 3.2 ppm and myo-inositol δ 3.5 ppm, were quantified using LCModel software (Stephen Provencher, Canada).

^1^H MR spectroscopy data were analyzed using LCModel software, version 6.3-0K (Stephen Provencher Inc, Canada) on a Linux workstation, and eddy current correction was performed. Spectra were phased and analyzed based on the “Tumor” basis of the LCModel software, which applied a linear combination of multiple spectra defined on the “Tumor” basis, by generating a Gaussian peak between a minimum and expected linewidth for each simulated peak, then applying a Lorentzian line-broadening to them all (LCModel User’s Manual). The resonances were quantified relative to the water signal. The Cramer-Rao lower bound (CRLB) value, which simultaneously accounts for both line width and signal-to-noise ratio, was calculated as an estimate of the error in metabolite quantification. MR spectra were excluded if the CRLB exceeded 20% for creatine (δ 3.0 and 3.9 ppm), choline (δ 3.2 ppm), myo-Inositol (δ 3.5 ppm), lipid methyl (δ 0.9 ppm) and lipid methylene (δ 1.3 ppm), and 30% for Glx (δ 2.2-2.4 ppm), lipid unsaturated (δ 2.0 ppm). A representative processing of the MR spectrum is shown in Figure 2.

For ^18^F-FDG PET/CT analysis, the SUV and MTV values of primary tumors were measured from attenuation-corrected ^18^F-FDG PET images using PMOD software (PMOD Technologies Ltd, Zurich, Switzerland). The boundaries were drawn sufficiently wide to include the primary tumor in axial, coronal, and sagittal ^18^F-FDG PET scans. An SUV threshold of 2.5 was used for the delineation of MTV [21, 36]. The contour around the target lesion inside the boundaries was automatically produced and the voxels were presenting SUV intensity > 2.5 within the contouring margin were incorporated to define MTV. TLG was calculated as the product of lesion mean SUV and MTV. The SUV, MTV, and TLG of the target lesions were automatically determined by the PMOD 3.2 software package (PMOD Technologies Ltd, Zurich, Switzerland).

### Outcome determination and statistical analysis

Local control was measured from the first day of chemoradiotherapy treatment to the time of local failure or the last follow-up. Local failure was determined by histological confirmation or by a serial increase in lesion size on follow-up imaging. The potential predicting local control parameters included age, tumor site, T status, N status, tumor size, creatine, choline, myo-inositol, Glx, lipid methyl (0.9 ppm), lipid methylene (1.3 ppm), and lipid unsaturated fatty acyls (2.0 ppm) on ^1^H MR spectroscopy as well as SUV_max_, MTV, and TLG on ^18^F-FDG PET/CT. The optimal cutoff values of these variables were determined using areas under the receiver operating characteristic curve (AUC). Correlations among the variables were assessed using Pearson’s correlation. ROC curve analysis was performed for age, tumor size, levels of metabolites on MR spectroscopy and ^18^F-FDG PET/CT parameters to get the optimal cut-off values of theses parameters to predict the occurrence of local recurrence within 2 years. These continuous variables were transformed into binary variables as “higher” and “equal or lower” than the cut-off values determined by ROC curve analysis. In univariate analysis, local control rates according to the associated parameters were plotted using the Kaplan-Meier method with the log-rank test for comparing the significance. Univariate Cox regression analysis was used to identify predictors of the two-year local control rates. Variables in univariate analyses were then entered into the multivariate Cox regression model, and a stepwise forward selection was used to identify significant independent predictors. A p value less than 0.05 was considered to be statistically significant. Data were analyzed using SPSS version 20.0.0 (IBM Corp. Released 2011. IBM SPSS Statistics for Mac, Version 20.0. Armonk, NY: IBM Corp.).

## Abbreviations

AUC: areas under the receiver operating characteristic curve
CRLB: Cramer-Rao lower bound
CT: computed tomography
DTPA: gadolinium diethylenetriaminepenta-acetic acid
^18^F-FDG PET/CT: [^18^F]-fluorodeoxyglucose positron emission tomography/CT
FOVs: fields of view
FWHM: full width at half maximum
Glx: glutamine and glutamate
Gy: Grays
^1^H MR: proton magnetic resonance
HNSCC: head and neck squamous cell carcinoma
mM: millimole
MRI: magnetic resonance imaging
MTV: metabolic tumor volume
OHSCC: oropharyngeal and hypopharyngeal squamous cell carcinoma
ppm: parts per million
PRESS: point-resolved spectroscopy
SUV: standardized uptake value
T: tesla
TLG: total lesion glycolysis
TSE: turbo spin echo
